# Caspase-2 inhibits mitochondrial respiration in colorectal adenocarcinoma cells

**DOI:** 10.1186/s12964-026-02671-z

**Published:** 2026-01-20

**Authors:** Chanin Sillapachaiyaporn, Maria A. Yapryntseva, Aygun R. Mamedova, Lina Abdelghany, Vladimir Gogvadze, Boris Zhivotovsky

**Affiliations:** 1 Unit of Toxicology, Institute of Environmental Medicine, Karolinska Institutet, Stockholm, 171 77 Sweden; 2 Engelhardt Institute of Molecular Biology, RAS, Moscow, 119991 Russia; 3 Faculty of Medicine, MV Lomonosov Moscow State University, Moscow, 119192 Russia; 4 Present Address: Department of Pharmacology and Toxicology, Faculty of Pharmacy, Tanta University, Tanta, 31527 Egypt

**Keywords:** Caspase-2, Mitochondria, Colorectal adenocarcinoma, SDH, TIMM/TOMM, P53

## Abstract

Knockout of caspase-2 in colorectal adenocarcinoma cells enhances mitochondrial oxygen consumption supported by succinate, a substrate of complex II. Additionally, caspase-2 knockout stimulates oxidative stress and promotes cell proliferation. The restriction of oxygen consumption by caspase-2 was attributed to the suppression of transcription of succinate dehydrogenase subunit B (SDHB), translocase of the outer mitochondrial membrane (TOMM), and the translocase of the inner mitochondrial membrane (TIMM). Caspase-2 knockout also increased the expression of the transcription factors p53 and c-Jun, which regulate the expression of mitochondria-related genes. Importantly, the catalytic activity of caspase-2 was dispensable for controlling mitochondrial respiration. In caspase-2 knockout mice, SDHA content remained unchanged, while SDHB and TIMM23 levels tended to increase, with TOMM20 showing the most prominent upregulation. Collectively, our findings uncover a novel molecular signaling pathway regulated by caspase-2 that may contribute to tumor growth suppression.

**Supplementary Information:**

The online version contains supplementary material available at 10.1186/s12964-026-02671-z.

## Introduction

Caspase-2, a highly conserved member of the caspase family, has been extensively studied for its role as a tumor suppressor during oncogenesis [1]. It plays a crucial role in protecting against cellular stress [2], and its loss has been associated with increased tumorigenesis in certain mouse models [1]. Studies have shown that *Casp2*^−/−^ mice exhibit elevated production of reactive oxygen species (ROS), including mitochondrially produced ROS (mtROS), along with impaired antioxidant responses [3, 4]. Additionally, the *Casp2*^−/−^ aged mice display increased activity of mitochondrial respiratory complex II, as observed in isolated liver mitochondria [5]. Increased mitochondrial respiratory activity has been suggested to correlate positively with cancers [6, 7]. However, the relationship between caspase-2 and the mitochondrial respiratory chain remains unclear.

Oxidative phosphorylation (OXPHOS) is a fundamental process by which mitochondria consume oxygen to generate energy through the electron transport chain (ETC). The ETC operates within the inner mitochondrial membrane and comprises four protein complexes: complex I, II, III, and IV [8]. Respiratory complex II, also known as succinate dehydrogenase (SDH), functions in both the ETC and the citric acid cycle. SDH is composed of four structurally distinct subunits: SDHA, SDHB, SDHC, and SDHD [9]. Mutations in SDH subunits can promote tumorigenesis [10–12]. In this study, we aimed to identify a potential connection between caspase-2 and mitochondrial activity using colorectal adenocarcinoma cells as a model, along with caspase-2 knockout mice.

## Materials and methods

### Cell cultures

Two colorectal adenocarcinoma cell lines, wild-type (SW620 WT) and caspase-2 knockout (SW620 *Casp2*^−/−^), previously generated in our laboratory [13], were cultured in Dulbecco’s Modified Eagle Medium (Gibco, Grand Island, NY, USA) supplemented with 100 U/mL penicillin, 100 µg/mL streptomycin (Sigma-Aldrich, Saint Louis, MO, USA), and 10% fetal bovine serum (Sigma-Aldrich). Cells were maintained in a humidified incubator at 37 °C with 5% CO_2_ and sub-cultured every 2–3 days using a trypsin–EDTA solution (Sigma-Aldrich). Cells in the exponential growth phase were used for the experiments.

### Animal experiments

*Casp2*^*−/−*^ mice were generated using CRISPR-Cas9 technology on an FVB/N background. CRISPR-Cas9 components targeting exon 5 of the caspase-2 gene were injected into the mouse zygotes, resulting in several mouse lines with different deletions in exon 5. The line carrying a 20-nucleotide deletion, which leads to mRNA degradation via the nonsense-mediated mRNA decay mechanism, was selected for experiments. Experimental groups included 2- to 3-month-old homozygous *Casp2*^del20^ males and females, as well as WT controls of both sexes. All animal procedures were approved by the Ethics Committee of Lomonosov Moscow State University (No. 3.6, approval date: May 16, 2024) and conducted following established guidelines for animal care. Animals were housed in a standard climate-controlled environment under a 12-h light–dark cycle at 22 ± 1 °C with 50 ± 10% humidity. Food and water were provided ad libitum.

### Oxygen consumption assay

Cells (3 × 10^6^ cells) were plated in 100-mm cell culture dishes and incubated for 2 days. After incubation, the cells were harvested by trypsinization and counted using trypan blue staining. Detached cells were pelleted by centrifugation at 1000 rpm for 5 min and resuspended in either fresh culture medium or a respiration buffer (150 mM KCl, 10 mM Tris, 5 mM K_2_HPO_4_, 1 mM MgCl_2_, pH 7.4) to analyze oxygen consumption in intact or plasma membrane-permeabilized cells, respectively. Oxygen consumption was monitored using a Clark-type oxygen electrode, as described previously [14]. Briefly, cells were transferred into the Oxygraph chamber, the lid was closed, and the decrease in the oxygen level was recorded over 3 min. Following this, 1 μM carbonyl cyanide chlorophenylhydrazone (CCCP) (Sigma-Aldrich) was injected using a Hamilton syringe to induce maximal mitochondrial respiration. To determine the activity of individual mitochondrial complexes, cells with digitonin-permeabilized plasma membranes were used. Digitonin was added at a final concentration of 0.01%. To assess mitochondrial respiratory complex I activity, 10 mM malonate (a complex II inhibitor) (Sigma-Aldrich) was applied before the addition of 10 mM pyruvate and 10 mM malate (complex I substrate) (Sigma-Aldrich). For complex II activity, 1 μM rotenone (a complex I inhibitor) (Sigma-Aldrich) and 8 mM succinate (a complex II substrate) (Sigma-Aldrich) were added. Complex III activity was assessed in the presence of rotenone and malonate to inhibit complexes I and II, along with 10 mM glycerol-3-phosphate (G3P; complex III substrate) (Sigma-Aldrich). Results were expressed as nmol O_2_/min/10^6^ cells.

In addition, oxygen consumption was assessed in liver homogenate from wild-type and caspase-2 knockout mice. Homogenates were prepared on ice to prevent tissue damage [15]. After euthanasia via isoflurane inhalation followed by cervical dislocation, the livers were washed with cold MSH buffer (210 mM mannitol, 70 mM sucrose, 5 mM Hepes, 1 mM EGTA, pH 7.4), weighed, cut with scissors, and gently homogenized on ice using a glass homogenizer with five to six manual strokes, adding 1 mL of medium per 1 g of tissue. The tissue suspension was centrifuged at 600 × *g* for 7 min at 4 °C to remove debris, and the resulting supernatant was used for subsequent experiments. Oxygen consumption was measured as described above, using a medium containing 0.1 M sucrose, 0.1 M KCl, 2 mM KH_2_PO_4_, and 5 mM Tris (pH 7.4). Succinate (5 mM) and rotenone (2 μM) were added to the homogenate in the chamber, and basal respiration was recorded. The uncoupler CCCP (1 μM) was then added to determine the maximal respiration rate. Protein concentration was measured using the Pierce™ BCA Protein Assay Kit (Thermo Scientific, Rockford, IL, USA). Data were analyzed using OriginPro software.

### MitoTracker assay

Cells were seeded into a 96-well black plate with a clear bottom at a density of 1 × 10^4^ cells/well and cultured in complete Dulbecco’s Modified Eagle Medium for 24 h. The cells were then double-stained with MitoTracker™ Green FM (500 nM) (Invitrogen, Thermo Fisher Scientific, Carlsbad, CA, USA) and Hoechst 34580 (5 μg/mL) (Sigma-Aldrich) for 45 min in a CO_2_ incubator at 37 °C. After staining, the cells were washed three times with Dulbecco’s phosphate-buffered saline (DPBS) and finally retained in this buffer. The fluorescence of MitoTracker™ Green FM and Hoechst 34580 was measured at an excitation/emission of 485/535 and 360/465 nm, respectively, using a microplate reader. The intensity of mitochondrial staining was normalized to the nuclear staining.

### Cell proliferation assay

The cells were plated into a 96-well plate at a density of 2 × 10^3^ cells/well. After culturing for 24, 48, 72, and 96 h, cell viability was assessed using the Cell Proliferation Kit II (XTT; Roche, Mannheim, Germany) according to the manufacturer’s instructions. Briefly, XTT reagent was added to the wells, and cells were incubated for an additional 4 h. Absorbance was measured at 490 nm with a reference wavelength of 650 nm using a microplate reader. Relative cell viability was calculated by comparing the absorbance at each time point to the 24-h value.

### H_2_DCFDA assay

The cells (1 × 10^4^ cells/well) were cultured in a 96-well black plate with a clear bottom and grown for 24 h. They were then incubated with H_2_DCFDA (10 μM) (Sigma-Aldrich) and Hoechst 34580 (5 μg/mL) in a CO_2_ incubator at 37 °C. After 45 min of incubation, the staining mixture was removed, and the cells were washed three times with DPBS. The fluorescence intensity of intracellular ROS (excitation/emission: 485/535 nm) and nuclear staining (excitation/emission: 360/465 nm) was measured using a microplate reader. The intensity of intracellular ROS staining was normalized to nuclear staining.

### MitoSOX assay

Cells were plated into a 96-well black plate with a clear bottom in the complete cell culture medium. After 24 h of incubation, the cells were stained with MitoSOX Red reagent (5 μM) (Invitrogen, Thermo Fisher Scientific) along with Hoechst 34580 (5 μg/mL) as a nuclear counterstain for 45 min. Following staining, cells were washed three times with DPBS, and the fluorescence intensity was measured using a microplate reader. MitoSOX fluorescence was measured at 540/590 nm (excitation/emission), and the Hoechst 34580 fluorescence at 360/465 nm (excitation/emission).

### Protein extraction and Western blotting

SW620 WT or SW620 *Casp2*^−/−^ cells were grown in 60-mm cell culture dishes at a density of 1 × 10^6^ cells per dish for 2 days. The cells were washed twice with cold DPBS, then lysed on ice using RIPA lysis buffer (Thermo Scientific) supplemented with a protease inhibitor cocktail (Roche). The cells were detached using a cell scraper, and the lysates were centrifuged at 14,000 rpm for 20 min. The protein concentration in the resulting supernatant was determined using the Pierce™ BCA Protein Assay Kit (Thermo Scientific) according to the manufacturer’s instructions. Sixty micrograms of protein were loaded onto a 12% SDS-PAGE gel and separated by electrophoresis. Proteins were transferred to a PVDF membrane (0.45-μm pore size), which was blocked with 3% milk in 0.1% TBST and then incubated with specific primary antibodies (1:1000 dilution) targeting proteins of interest. The following antibodies were used: anti-caspase-2 mouse monoclonal antibody (Cat. No. 611023; BD Biosciences, San Diego, CA, USA); anti-SDHA (D6J9M) XP® rabbit monoclonal antibody (Cat. No. 11998); anti-SDHB (E3H9Z) XP® rabbit monoclonal antibody (Cat. No. 92649), anti-TIMM23 (E1Q7L) rabbit monoclonal antibody (Cat. No. 34822), anti-GAPDH (14C10) rabbit monoclonal antibody (Cat. No. 2118), and anti-c-Jun rabbit monoclonal antibody (Cat. No. 9165) (all from Cell Signaling Technology, Danvers, MA, USA); anti-TOMM20 rabbit monoclonal antibody (Cat. No. ab186735) and anti-TOMM40 rabbit monoclonal antibody (Cat. No. ab185543) (both from Abcam, Cambridge, UK); anti-TIMM21 mouse polyclonal antibody (Cat. No. PA5-100,224; Invitrogen, Thermo Fisher Scientific); anti-p53 (FL-393) rabbit polyclonal antibody (Cat. No. SC-6243; Santa Cruz Biotechnology, Dallas, TX, USA); anti-JNK/SAPK-1/2 mouse monoclonal antibody (Cat. No. AT-7041; MBL International, Schaumburg, IL, USA). After washing, the membranes were incubated with IRDye secondary antibodies (1:5000 dilution), and protein bands were visualized using the Odyssey CLx Imager (LI-COR Biosciences, Lincoln, NE, USA). Band densitometry was performed by using Image Studio™ Lite software, with protein levels normalized to GAPDH as a loading control.

### Silencing of caspase-2 in SW620 WT cells

SW620 WT cells (1 × 10^6^ cells) were seeded into 60-mm cell culture dishes and incubated overnight. The cells were then transfected with siRNA targeting caspase-2 (siRNA-*Casp2*) or a negative control siRNA (siRNA-NC) for 72 h using Lipofectamine™ 3000 reagent (Thermo Fisher Scientific). After transfection, proteins were extracted from the cells, and western blot analysis was performed as described above.

### RNA extraction and RT-qPCR

SW620 WT or SW620 *Casp2*^−/−^ cells were seeded into a 6-well plate at a density of 5 × 10^5^ cells per well and cultured for 2 days. RNA was extracted using TRIzol™ reagent (Invitrogen, Thermo Fisher Scientific). RNA concentration was measured using a NanoDrop ONE spectrophotometer (Thermo Fisher Scientific). The extracted RNA was reverse-transcribed into cDNA using a High-Capacity cDNA Reverse Transcription Kit (Applied Biosystems™, Thermo Fisher Scientific Baltics UAB, Vilnius, Lithuania). qPCR amplification of the cDNA templates was performed using a PowerTrack™ SYBR Green Master Mix (Applied Biosystems™, Thermo Fisher Scientific Baltics UAB) on a QuantStudio™ 5 Real-Time PCR System (Applied Biosystems™, Thermo Fisher Scientific, Waltham, MA, USA). Specific primers for *Casp2, Sdha, Sdhb, Timm21, Timm23, Tomm20, Tomm40, Cat, Nqo1, Tp53*, and *Actb* genes are shown in Table 1. The relative gene expression was calculated by using the delta-delta C_t_ method.

**Table 1:** List of primer pairs used for qPCR

Gene	Primer	Nucleotide sequence (5’ – > 3’)
*Casp2*	Forward	GAACACTCCCTAGACAATAAAG
	Reverse	AGCGAAATTCCAGTTCTTTC
*Sdha*	Forward	GGAACAAGAGGGCATCTGCT
	Reverse	CCGTCATGTAGTGGATGGCA
*Sdhb*	Forward	CTGACACGCCAGAAGTAGCA
	Reverse	CATGGGTTCCTGTGCATCCT
*Timm21*	Forward	GCTATGGGGAGGTGACAAGG
	Reverse	ACCCCACATCCCATGATTCC
*Timm23*	Forward	AGCAGCTGGAACCATGACAG
	Reverse	GAGATGGCTCCCCATTCAACA
*Tomm20*	Forward	CCACCAGTGTTCCAGATGCT
	Reverse	AGCGCTGATATCTCCCATATTGT
*Tomm40*	Forward	ATATGGTGGGAAGCTGGCAC
	Reverse	AGGCCAAAGCCACACTGAAA
*Cat*	Forward	TCTCACCAAGGTTTGGCCTC
	Reverse	CGGCCCTGAAGCATTTTGTC
*Nqo1*	Forward	GCTCACCGAGAGCCTAGTTC
	Reverse	CCACCACCTCCCATCCTTTC
*Tp53*	Forward	AGGAAATTTGCGTGTGGAGTAT
	Reverse	TCCGTCCCAGTAGATTACCACT
*Actb*	Forward	GCTGTGCTATCCCTGTACGC
	Reverse	GAGGGCATACCCCTCGTAGA

### Prediction of transcription factors and protein–protein interaction analysis

The nucleotide sequences of the promoter regions, ranging from −499 to + 100 relative to the transcription start site, for the *Timm21, Timm23, Tomm20, Tomm40*, and *Sdhb* genes were retrieved from the Eukaryotic Promoter Database (https://epd.expasy.org; accessed on 29 April 2024). Transcription factors potentially binding to these promoter regions were predicted using the PROMO online tool (https://alggen.lsi.upc.es; accessed on April 29, 2024). The predicted transcription factors were then analyzed for possible protein–protein interactions with caspase-2 using the STRING database (https://string-db.org; accessed on April 29, 2024).

### Silencing p53 in SW620 *Casp2*^−/−^ cells

Silencing of p53 in SW620 *Casp2*^−/−^ cells was performed using two methods that mediate the RNA interference effect – siRNA and short hairpin RNA (shRNA). For siRNA-mediated *TP53* knockdown, SW620 *Casp2*^−/−^ cells (5 × 10^5^ cells/well) were plated into a 6-well plate and incubated overnight. The cells were then transfected with siRNA-Tp53 or siRNA-NC for 72 h using Lipofectamine™ 3000 reagent (Thermo Fisher Scientific). Transfection efficiency was assessed by evaluating *Tp53* gene expression using RT-qPCR.

SW620 *Casp2*^−/−^ cells with *TP53* knockdown using shRNA technology were obtained as previously described [16]. Briefly, cells were plated into a 6-well plate overnight. Then, cells were transduced with lentiviral shRNA constructs targeting *TP53*, which were kindly provided by Dr. Peter Chumakov, and selected with Puromycin (Gibco) and Geneticin (Gibco). Knockdown efficiency was confirmed by evaluating the p53 level by Western blotting.

### Overexpression of mutant (S384A) or WT caspase-2 in SW620 *Casp2*^−/−^ cells

SW620 *Casp2*^−/−^ cells were cultured overnight in 60-mm cell culture dishes at a density of 5 × 10^5^ cells per dish. The cells were transfected with pESG-IBA103 (empty vector), pESG-IBA103-caspase-2 (WT), or pESG-IBA103-caspase-2 (S384A) plasmids using Lipofectamine™ 3000 reagent (Thermo Fisher Scientific) for 72 h. Overexpression was confirmed by western blotting.

### Statistical analysis

Data are presented as mean ± standard deviation (SD) from at least three independent experiments. Statistical analysis was performed using GraphPad Prism software. Differences between the two groups were evaluated using Student’s t-test, while comparisons among more than two groups were assessed using one-way analysis of variance followed by Dunnett’s post hoc test. *P*-values of < 0.05 were considered statistically significant.

## Results

### Caspase-2 controls mitochondrial respiration in colorectal adenocarcinoma cells

**Fig. 1:**
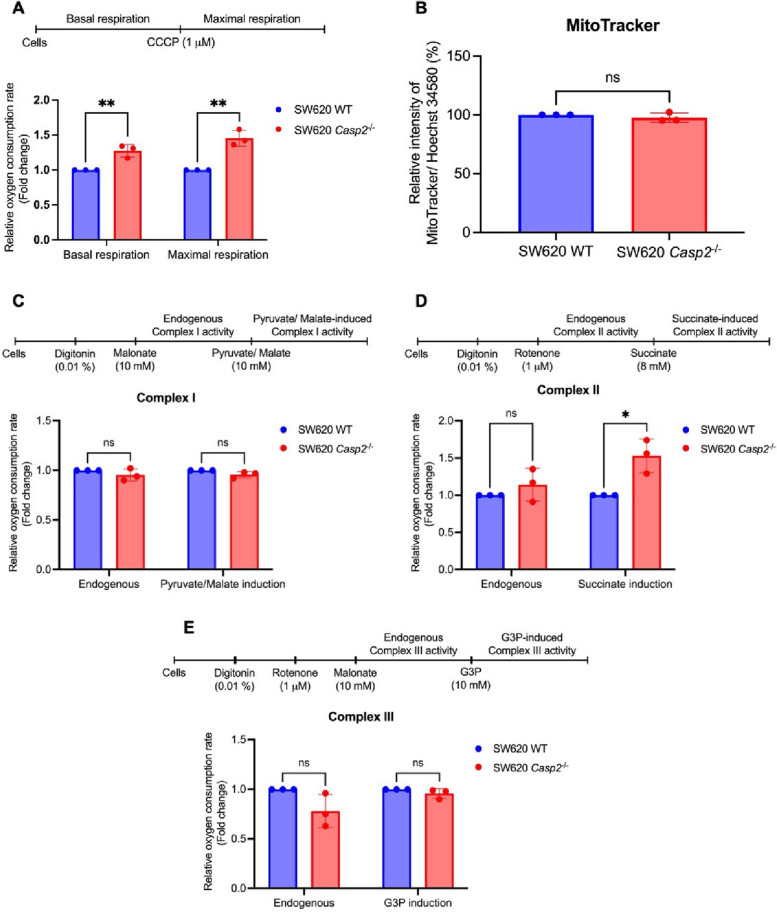
Regulation of mitochondrial respiration by caspase-2 in colorectal adenocarcinoma cells. **A** Basal and CCCP-uncoupled mitochondrial respiration in SW620 WT and SW620 *Casp2*^−/−^ cells. **B** Mitochondrial content in SW620 WT and SW620 *Casp2*^−/−^ cells. **C–E** Respiratory activity of various complexes of the mitochondrial respiratory chain in SW620 WT and SW620 *Casp2*^−/−^ cells. Results are shown as the mean ± SD of three independent experiments. *P < 0.05, **P < 0.01, ns = not significant

The role of caspase-2 in regulating mitochondrial function in colorectal adenocarcinoma cells was initially assessed by measuring mitochondrial oxygen consumption in intact cells. Cells lacking caspase-2 exhibited a significant increase in both basal and CCCP-uncoupled mitochondrial respiration compared with wild-type cells, approximately 20% and 40% higher, respectively (Fig. 1A). To determine whether this increase in respiration was not caused by stimulation of mitochondrial biogenesis, the number of mitochondria in SW620 WT and SW620 *Casp2*^−/−^ cells was assessed using the MitoTracker™ reagent. As shown in Fig. 1B, the relative number of mitochondria did not differ significantly between the two cell lines. These results suggest that caspase-2 restricts mitochondrial respiration without altering mitochondrial abundance. Furthermore, we examined the activity of individual mitochondrial respiratory complexes in SW620 WT and SW620 *Casp2*^−/−^ cells using digitonin-permeabilized cells.

As shown in Fig. 1C-E, knockout of caspase-2 significantly increased oxygen consumption in the presence of succinate, a complex II substrate, whereas complexes I and III showed no significant changes.

### Caspase-2 knockout stimulated oxidative stress and cell proliferation in colorectal adenocarcinoma cells

**Fig. 2:**
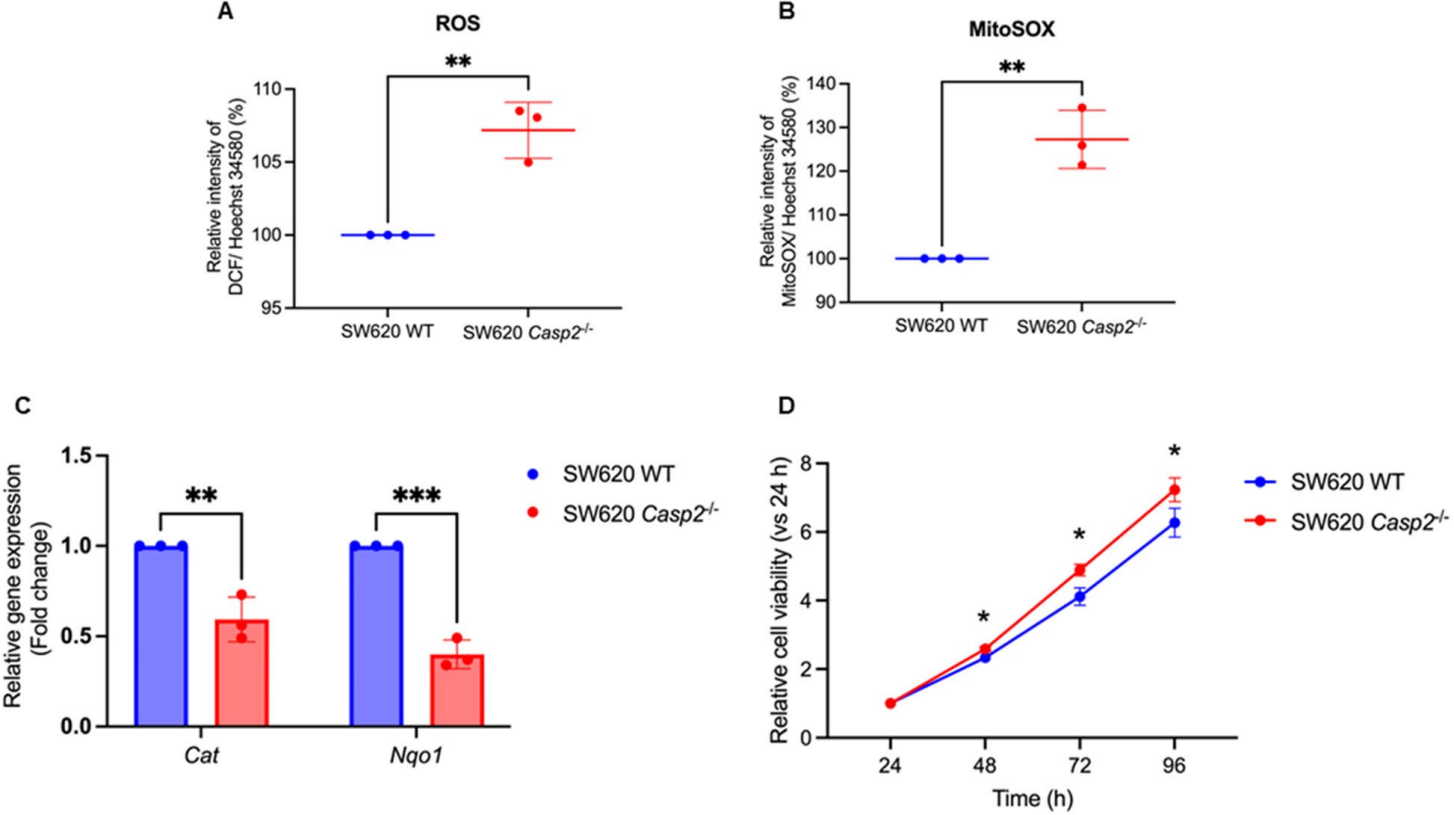
Caspase-2 regulation of oxidative stress responses and cell proliferation in colorectal adenocarcinoma cells. **A**, **B** Production of ROS and mtROS, respectively. **C** Relative expression of antioxidant-associated genes *Cat* and *Nqo1*. **D** Relative cell viability at 24, 48, 72, and 96 h of culture in SW620 WT and SW620 *Casp2*^*−/−*^ cells. Results are shown as the means ± SD of three independent experiments. *P < 0.05, **P < 0.01, ***P < 0.001, ns = not significant

In addition to producing ATP, mitochondria generate ROS as byproducts of oxygen consumption [17, 18]. To assess oxidative stress, intracellular ROS levels in SW620 WT and *Casp2*^−/−^ cells were measured using the H_2_DCFDA assay. SW620 *Casp2*^*−/−*^ cells produce approximately 10% more ROS than wild-type cells do (Fig. 2A). To specifically evaluate mitochondrial superoxide production, the superoxide indicator MitoSOX™ Red was used. As shown in Fig. 2B, caspase-2 knockout significantly increased mitochondrial superoxide generation. Gene expression analysis further revealed that mRNA levels of the antioxidant-related genes *Cat* and *Nqo1* were downregulated in *Casp2*^−/−^ cells (Fig. 2C). Because mtROS generation can influence cellular signaling, induce DNA instability, and affect proliferation, the cell proliferation was assessed through analysis of factors known to contribute to tumorigenesis [19, 20]. Caspase-2-deficient cells exhibited significantly increased proliferation from day 2 to 4 of culturing compared with WT cells (Fig. 2D). Taken together, these results suggest that caspase-2 plays a role in limiting mtROS production and proliferation in SW620 cells.

### Caspase-2 decreases mitochondria-related gene expression and protein content

**Fig. 3:**
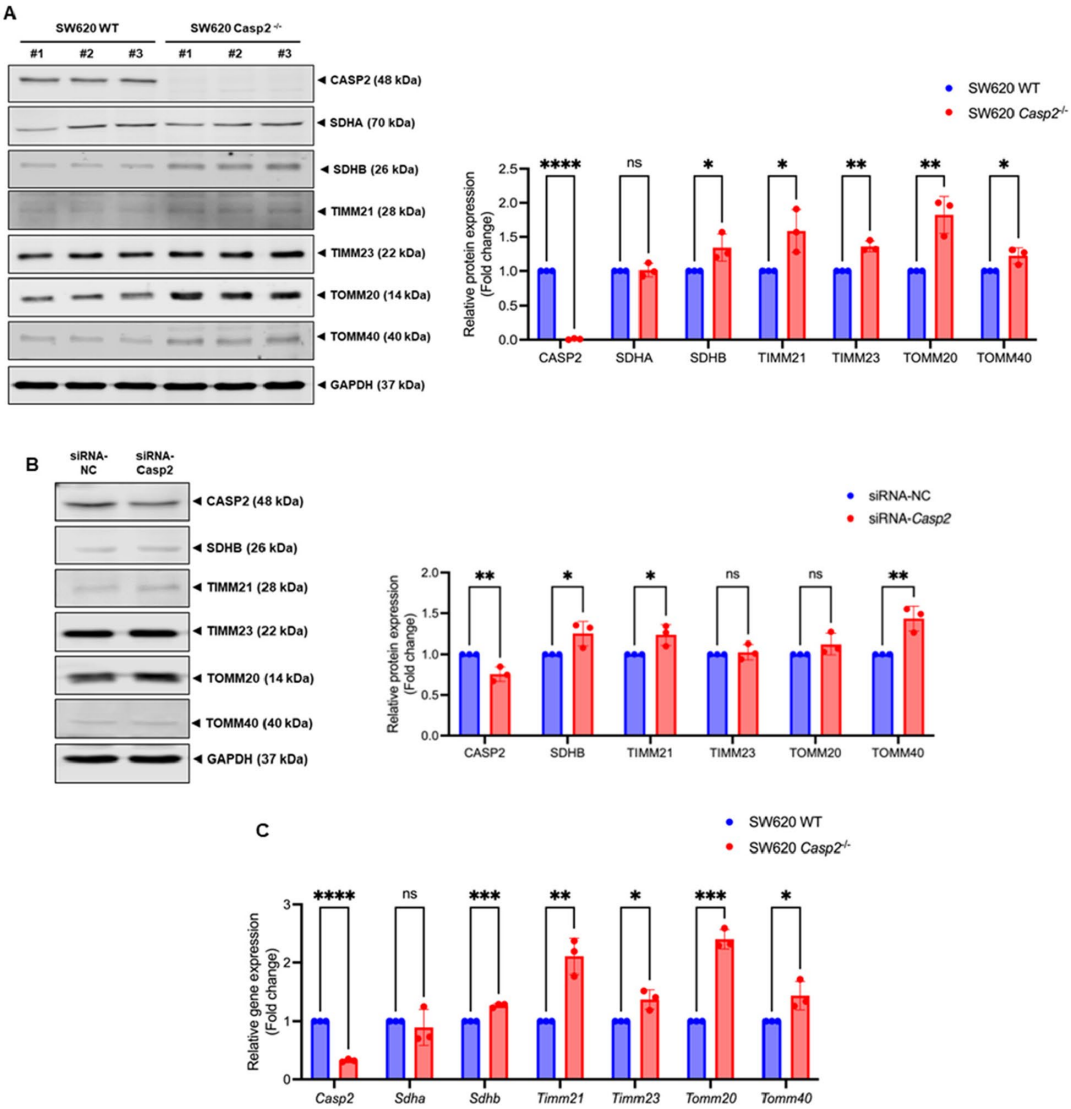
Effects of caspase-2 on mitochondria-related gene expression and protein content in colorectal adenocarcinoma cells. **A** Representative immunoblots and relative protein levels of CASP2, SDHA, SDHB, TIMM21, TIMM23, TOMM20, and TOMM40 in SW620 WT and SW620 *Casp2*^*−/−*^ cells. **B** Representative immunoblots and relative protein levels of CASP2, SDHB, TIMM21, TIMM23, TOMM20, and TOMM40 in SW620 WT cells transfected with siRNA targeting *Casp2* or negative control (NC). **C** Relative expressions of mitochondria-associated genes: *Casp2, Sdha, Sdhb, Timm21, Timm23, Tomm20*, and *Tomm40* in SW620 WT and SW620 *Casp2*^*−/−*^ cells. Results are shown as the means ± SD of three independent experiments. *P < 0.05, **P < 0.01, ***P < 0.001, ****P < 0.0001, ns = not significant

To explain the observed increase in succinate-supported respiration in *Casp2*^−/−^ cells, the protein content of mitochondrial respiratory complex II was examined. Immunoblot analysis showed that the level of SDHB was significantly elevated in SW620 *Casp2*^−/−^ cells, while the level of the SDHA subunit remained unchanged (Fig. 3A). These results suggest that caspase-2 may reduce mitochondrial respiratory complex II activity by downregulating SDHB content.

SDHB is a nuclear-encoded protein that must be translocated into the mitochondria [21]. Therefore, the level of mitochondrial protein import-related proteins, including the translocase of the inner mitochondrial membrane (TIMM) and the outer mitochondrial membrane (TOMM) proteins, was examined. In SW620 *Casp2*^*−/−*^ cells, the protein levels of TIMM21, TIMM23, TOMM20, and TOMM40 were significantly increased as compared with SW620 WT cells (Fig. 3A). To confirm these findings, siRNA technology was used to generate the caspase-2 knockdown cells. Consistent with the knockout model, caspase-2 knockdown cells also showed increased levels of SDHB, TIMM21, and TOMM40 proteins (Fig. 3B). Additionally, gene expression analysis revealed that *Sdhb, Timm21, Timm23, Tomm20*, and *Tomm40* were significantly upregulated in SW620 *Casp2*^*−/−*^ cells (Fig. 3C). A similar increase in mitochondria-related protein content was observed in HCT116 colorectal cancer cells lacking caspase-2 (Supplementary data, Figure S1). These results suggest that caspase-2 suppresses the expression of these mitochondria-related proteins at both the gene and protein levels.

### Caspase-2 suppresses the expression of transcription factors p53 and c-Jun, and subsequently downregulates transcriptional expression of mitochondria-related genes

**Fig. 4:**
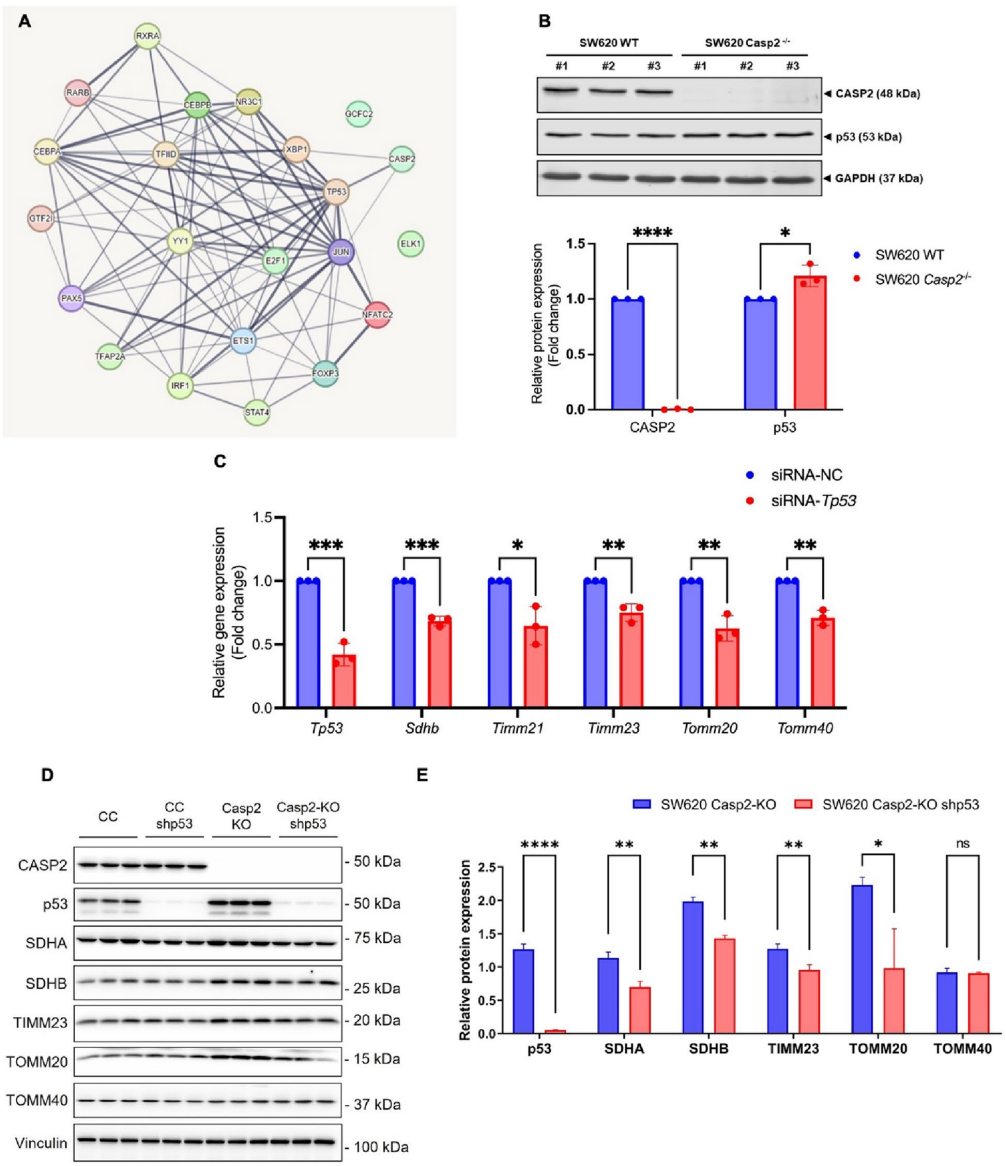
Identification of caspase-2-downstream proteins regulating mitochondria-related gene transcription. **A** Protein–protein interaction network of the predicted transcription factors and caspase-2, based on the STRING database. **B** Representative immunoblots and relative protein levels of CASP2 and p53 in SW620 WT and SW620 *Casp2*^*−/−*^ cells. **C** Relative gene expression of *Tp53, Sdhb, Timm21, Timm23, Tomm20*, and *Tomm40* in SW620 *Casp2*.^*−/−*^ cells transfected with siRNA targeting *Tp53* or negative control (NC). **D** Representative immunoblots and (**E**) relative protein levels of CASP2, p53, SDHA, SDHB, TIMM23, TOMM20, and TOMM40 in SW620 Casp2-KO and SW620 Casp2-KO + shp53 cells. Results are shown as the mean ± SD of three independent experiments. *P < 0.05, **P < 0.01, ***P < 0.001, ****P < 0.0001

To investigate the transcriptional regulation of mitochondria-related genes, potential transcription factors that could bind to the promoter regions of *Sdhb*, *Timm21*, *Timm23*, *Tomm20*, and *Tomm40* were identified using the PROMO online analysis tool (https://alggen.lsi.upc.es). According to the database, 24 transcription factors were predicted to bind to the promoter regions of these genes: FOXP3, YY1, GR-α, IRF-1, TFIID, NF-AT1, C/EBP-β, C/EBP-α, TFII-I, GR, GR-β, Pax-5, p53, ENKTF-1, STAT4, c-Ets-1, E2F-1, RXR-α, GCF, AP-2-αA, XBP-1, c-Jun, Elk-1, and RAR-β. The predicted transcription factors were then analyzed for potential interaction with caspase-2 using the STRING database (https://string-db.org). Among them, three, XBP1, p53, and c-Jun, have been previously reported to interact with caspase-2 (Fig. 4A). According to earlier studies, XBP1 is recognized as an upstream regulator of caspase-2 [22–24]. Therefore, we focused on the downstream targets of p53 and c-Jun.

The protein levels of p53 and c-Jun in SW620 cells were further evaluated. The level of p53 was significantly increased in SW620 *Casp2*^*−/−*^ cells compared with WT cells (Fig. 4B). To confirm the role of p53 in the transcriptional regulation of mitochondria-related genes, p53 knockdown was performed using the siRNA approach. The expression of *Sdhb, Timm21, Timm23, Tomm20*, and *Tomm40* was significantly decreased in p53-silenced SW620 *Casp2*^*−/−*^ cells compared with non-transfected cells (Fig. 4C). The content of the corresponding proteins changed in the same way (Fig. 4D-E). These results support the role of p53 as a mediator of the caspase-2 signaling pathway involved in regulating mitochondria-related gene expression.

 Further, the relationship between caspase-2 and c-Jun in SW620 cells was investigated. Consistent with the protein–protein interaction analysis, SW620 *Casp2*^*−/−*^ cells showed significant upregulation of JNK1 and c-Jun protein levels (Supplementary Data, Figure S2). This finding suggests that caspase-2 may suppress the JNK1/c-Jun signaling pathway, thereby downregulating the expression of mitochondria-associated genes.

### Catalytic activity of caspase-2 is not required for regulating mitochondrial respiration

**Fig. 5:**
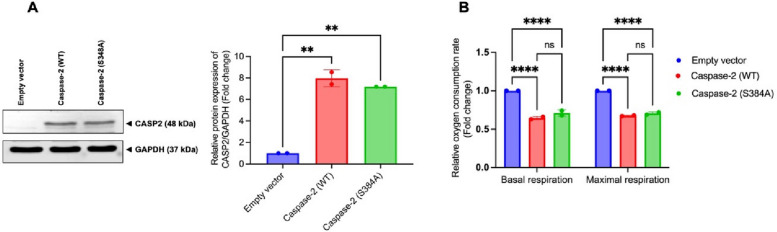
Effects of wild-type and catalytically inactive mutant (S384A) of caspase-2 on mitochondrial respiration in colorectal adenocarcinoma cells. **A** Overexpression of caspase-2 WT and mutant (S384A) in SW620 *Casp2*^−/−^ cells (**B**) Basal and CCCP-uncoupled mitochondrial respiration in caspase-2 WT- or mutant (S384A)-overexpressing SW620 *Casp2*^−/−^ cells. Results are shown as the mean ± SD of two independent experiments. **P < 0.01, ****P < 0.0001, ns = not significant

To determine whether the catalytic activity of caspase-2 is necessary for the suppression of mitochondrial respiration, both WT and catalytically inactive mutant (S384A) caspase-2 [13] were re-expressed in SW620 *Casp2*^−/−^ cells (Fig. 5A). Mitochondrial oxygen consumption was then measured in these overexpressing cells and compared to that in empty vector-transfected controls. As shown in Fig. 5B, both WT and mutant caspase-2-expressing SW620 *Casp2*^−/−^ cells exhibited significantly lower basal and uncoupled oxygen consumption compared with empty vector-transfected cells. Notably, no significant difference in mitochondrial oxygen consumption was observed between cells re-expressing WT or mutant caspase-2. These results indicate that the suppression of mitochondrial respiration by caspase-2 does not depend on its enzymatic activity.

### Mitochondrial respiration in ***Casp2***^***−/−***^ mice.

To confirm the effects of *Casp2*^*−/−*^ *in vivo*, a *Casp2*^*−/−*^ mouse model was generated. The absence of caspase-2 was verified at both the mRNA and protein levels. Young animals (2–3 months old) of both sexes were used for the experiments.

**Fig. 6:**
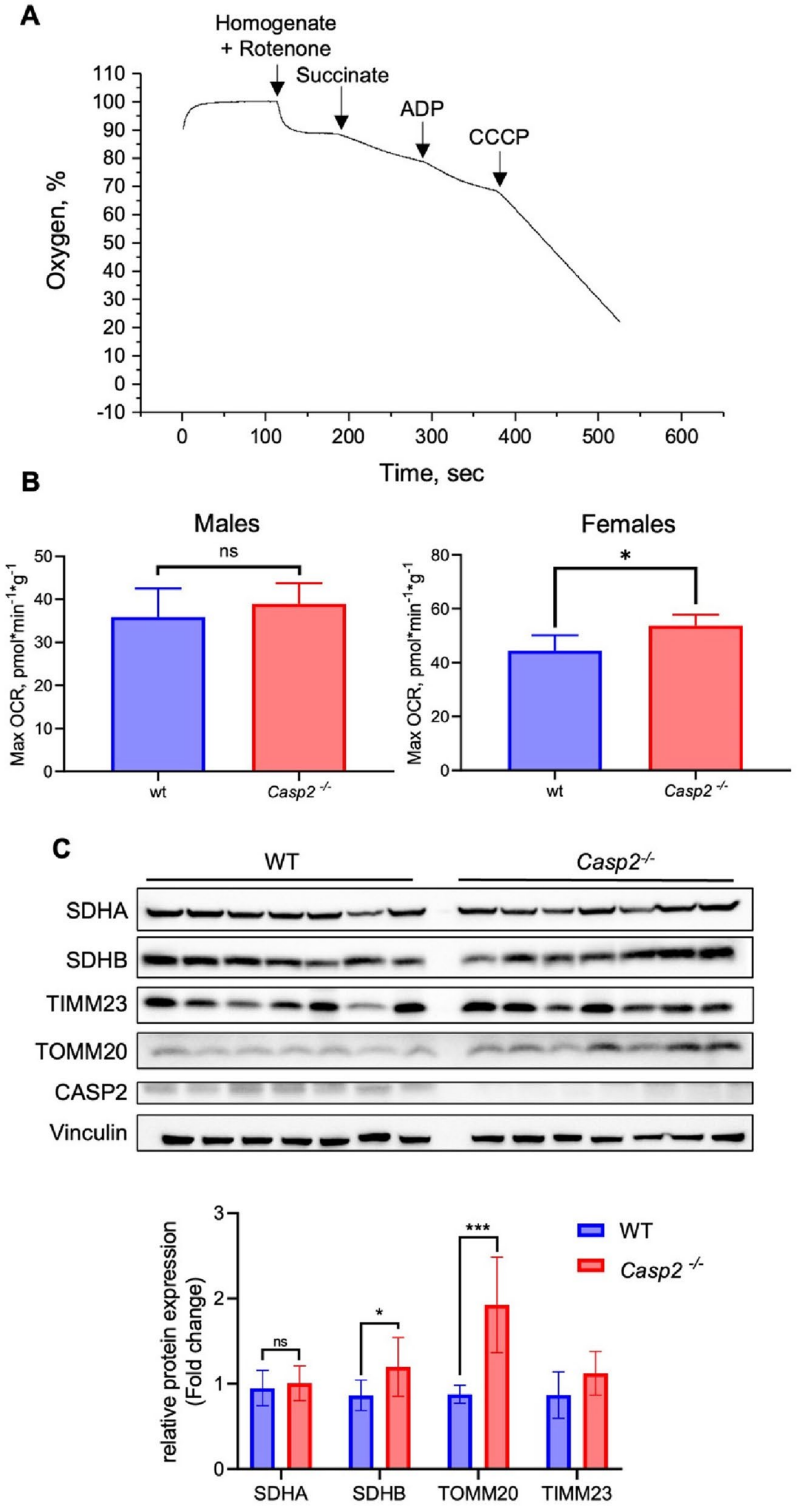
Effects of caspase-2 on liver homogenate oxygen consumption and respiratory chain protein levels in mice. **A** Representative trace of oxygen consumption in mouse liver homogenate. **B** Maximal respiration levels in WT and *Casp2*^*−/−*^ mice. **C** Representative immunoblots and relative protein levels of SDHA, SDHB, TIMM23, TOMM20, and CASP2 in liver samples from WT and *Casp2*.^*−/−*^ female mice. Data are presented as mean ± SD, *P < 0.05, **P < 0.001

Analysis of oxygen consumption in liver homogenates revealed that in female mice, caspase-2 knockout enhanced maximal respiration (in the presence of CCCP), consistent with previous findings in cell culture. By contrast, no significant difference in respiration was observed between the *Casp2*^*−/−*^ and wild-type male mice (Fig. 6A and B).

Next, the levels of select mitochondrial proteins in the liver tissue were assessed by immunoblotting. SDHA, SDHB, TOMM20, and TIMM23 protein levels were compared between groups of seven WT and *Casp2*^*−/−*^ female mice. In line with the SW620 cell data, caspase-2 deficiency did not alter SDHA levels, while SDHB and TIMM23 levels showed a tendency to increase in the knockout group (Fig. 6C). The most prominent upregulation was observed for TOMM20.

## Discussion

Comparative analysis of mitochondrial activity between WT and *Casp2*^*−/−*^ colorectal adenocarcinoma cells revealed that caspase-2 restrains mitochondrial respiration, particularly complex II activity. A previous study on caspase-2 knockout mice reported that the metabolic profile of young *Casp2*^*−/−*^ mice resembled that of aged wild-type mice [5]. Assessment of individual mitochondrial complexes showed decreased activity of both citrate synthase and complex III in aged and young *Casp2*^*−/−*^ mice compared with young wild-type mice. At the same time, the authors observed a trend toward increased spare respiratory capacity in *Casp2*^*−/−*^ mouse embryonic fibroblasts (*P* = 0.08), although the earlier study found no difference in mitochondrial function following caspase-2 loss in primary mouse embryonic fibroblasts [3]. In our experiments using SW620 cells, only complex II activity was significantly increased in *Casp2*^*−/−*^ cells, suggesting that caspase-2 knockout may affect mitochondrial function differently depending on the tissue type. Notably, the SW620 cell line, used in our study, was derived from a 51-year-old male patient, who would be considered an aged individual [25], aligning our findings with previous reports on the age-dependent effects of caspase-2 on mitochondrial respiratory complexes activity. Additionally, the increase in oxygen consumption observed in *Casp2*^*−/−*^ cells was not due to an increase in mitochondrial abundance, consistent with earlier findings showing that *Casp2*^*−/−*^ hepatocytes did not exhibit changes in mitochondrial DNA copy number compared to WT cells [5].

In addition, caspase-2 knockout impairs antioxidant activity, leading to the accumulation of ROS. These effects are mediated by the reduced expression of antioxidant enzymes such as glutathione peroxidase, catalase, and superoxide dismutase through the FoxO1/FoxO3a signaling pathway [2, 3, 26]. Consistent with these studies, our data also show suppressed antioxidant activity, specifically the downregulation of *Cat* and *Nqo1* gene expression. Increased ROS is a double-edged sword; while it can induce apoptotic cell death, it can also promote tumor cell proliferation [27]. In the current study, elevated ROS levels in *Casp2*^*−/−*^ cells were associated with enhanced cell proliferation. These findings support the role of caspase-2 as a tumor suppressor, consistent with previous reports [1], in addition to its established function in apoptosis.

Here, we revealed that the gene expression and protein level of SDHB were increased in SW620 *Casp2*^*−/−*^ cells. The role of SDHB and other SDH subunits in carcinogenesis is not fully understood. For instance, breast tumor tissues from patients showed high expression of SDHA and SDHB, while their expression was lower in stromal cells (normal controls) [11]. However, other studies reported decreased SDHB expression in hepatocellular carcinoma and breast cancer cells, which promoted tumor malignancy by shifting metabolism from aerobic respiration to glycolysis (the Warburg effect), thereby enhancing tumor cell proliferation and metastasis [10, 12]. Moreover, in colorectal cancer cell lines (SW480, SW620, SW116, and HT-29), SDHB expression at both gene and protein levels was lower compared with non-malignant NCM460 cells. Interestingly, knockdown of SDHB in colorectal adenocarcinoma cells did not show a notable effect on cell proliferation [28]. By contrast, our current study demonstrated that caspase-2 knockout in colorectal cancer cells led to increased SDHB expression and promoted tumor cell growth. Taken together, these findings suggest that caspase-2 mediates multiple signaling pathways beyond the regulation of SDHB alone, highlighting the need for further investigation into other mechanisms influenced by caspase-2. Notably, in addition to its role in mitochondrial respiration, SDH also regulates various succinate-dependent signaling pathways, including those involved in DNA methylation, inflammation, and cell fate decisions [29].

As we found, among all mitochondrial respiratory complexes, only complex II (SDH) showed a significant increase in function in SW620 *Casp2*^*−/−*^ cells. SDH consists of four nuclear-encoded subunits that must independently translocate to the mitochondria via the TIMM and TOMM complexes and assemble into a mature protein [21, 30]. Therefore, caspase-2 may influence SDH activity by affecting SDH maturation-related proteins, such as SDH assembly factors [31], representing one possible mechanism by which caspase-2 alters OXPHOS. Additionally, the observed upregulation of TIMM and TOMM gene and protein expression in SW620 *Casp2*^*−/−*^ cells suggests that caspase-2 may regulate their transcription. It has been shown that the transcription factor NRF2 can bind to the promoter region and activate *TOMM20* gene transcription [32]. Furthermore, increased NRF2 protein expression has been reported in *Casp2*^*−/−*^ cells [2], suggesting a potential link between caspase-2, NRF2, and TOMM20. Our data suggest that the promoter regions of the *TIMM, TOMM*, and *SDHB* genes could be regulated by several transcription factors, including p53.

p53 is a well-known gene and protein recognized for its tumor-suppressive activity. Several studies have shown that reduced p53 expression is associated with tumorigenesis [33–35]. Our findings demonstrate that SW620 *Casp2*^*−/−*^ cells exhibit higher p53 protein levels than SW620 WT cells do. The deficiency of caspase-2 alongside elevated p53 in SW620 cells may contribute to enhanced cancer cell proliferation. Previous studies have also reported increased *p53* gene expression and protein levels in *Casp2*^*−/−*^ cells [3, 36]. Notably, p53 has been identified as a negative regulator of ferroptosis in colorectal cancer cells by inhibiting dipeptidyl-peptidase-4 (DPP4), an enzyme involved in lipid peroxidation and ferroptotic cell death [37]. Based on this, caspase-2 may promote ferroptosis by suppressing the p53/DPP4 signaling pathway. By contrast, a recent study reported that caspase-2 can inhibit ferroptotic cell death in p53-mutant lung and esophageal cancer cells by preventing the degradation of glutathione peroxidase 4, a key anti-ferroptotic enzyme [38]. These seemingly contradictory roles may be explained by differences in p53 genotype and specific cancer cell context.

Furthermore, in SW620 *Casp2*^*−/−*^ cells, the expression of JNK1 and the level of c-Jun were increased. This finding is consistent with previous reports on JNK and c-Jun upregulation in caspase-2 deficiency models [36, 39]. Activation of the JNK stress-response pathway was significantly elevated in *Casp2*^*−/−*^ mice, rendering them more susceptible to chemically induced liver cancer development [36]. Additionally, our computational analysis revealed that the c-Jun transcription factor could bind to the promoter regions of *Sdhb, Timm21, Timm23, Tomm20*, and *Tomm40*, suggesting a regulatory role in their transcription. JNK activation has also been shown to increase ROS production, while silencing JNK slightly reduced basal oxygen consumption in HeLa cells [40]. Taken together, these findings suggest a possible molecular mechanism by which caspase-2 regulates OXPHOS through the JNK signaling pathway.

Experiments with mice confirmed the involvement of caspase-2 in regulating complex II of the mitochondrial respiratory chain. As previously noted, age-dependent mitochondrial dysfunction and increased oxidative stress have been observed in *Casp2*^*−/−*^ mice, particularly in older males [3–5, 41]. However, young male *Casp2*^*−/−*^ mice have shown altered basal metabolism and enhanced whole-body carbohydrate utilization [42], suggesting a potential link between caspase-2 and metabolic function. In the present study, however, we did not observe any significant changes in mitochondrial respiration in young male *Casp2*^*−/−*^ mice.

Sex influences the metabolic profile of various tissues, including the liver. Analysis of oxygen consumption supported by different respiratory chain complexes across tissues has shown that both age and sex affect mitochondrial respiration. Notably, age-dependent alterations in respiratory function differ between sexes [43]. One key factor underlying this difference is estrogen, which plays a significant role in lipid turnover and carbohydrate metabolism. A peak of the estrogen levels in females is around 6 months of age and declines thereafter, while in males, estrogen levels remain relatively stable over time, accompanied by a gradual decline in testosterone. This hormonal profile may cause the metabolic characteristics of aged males to resemble those of young females [44]. Sex-specific differences in glucose and lipid metabolism have also been reported in caspase-2-deficient mice [45], which could help explain the sex-specific differences observed in our study, specifically, the increased mitochondrial respiration seen in female *Casp2*^*−/−*^ mice.

Because the consequences of caspase-2 knockout may vary depending on the age and sex of the mice, targeting caspase-2 for cancer therapy could elicit different responses based on these factors. This possibility warrants further investigation.

## Conclusion

**Fig. 7:**
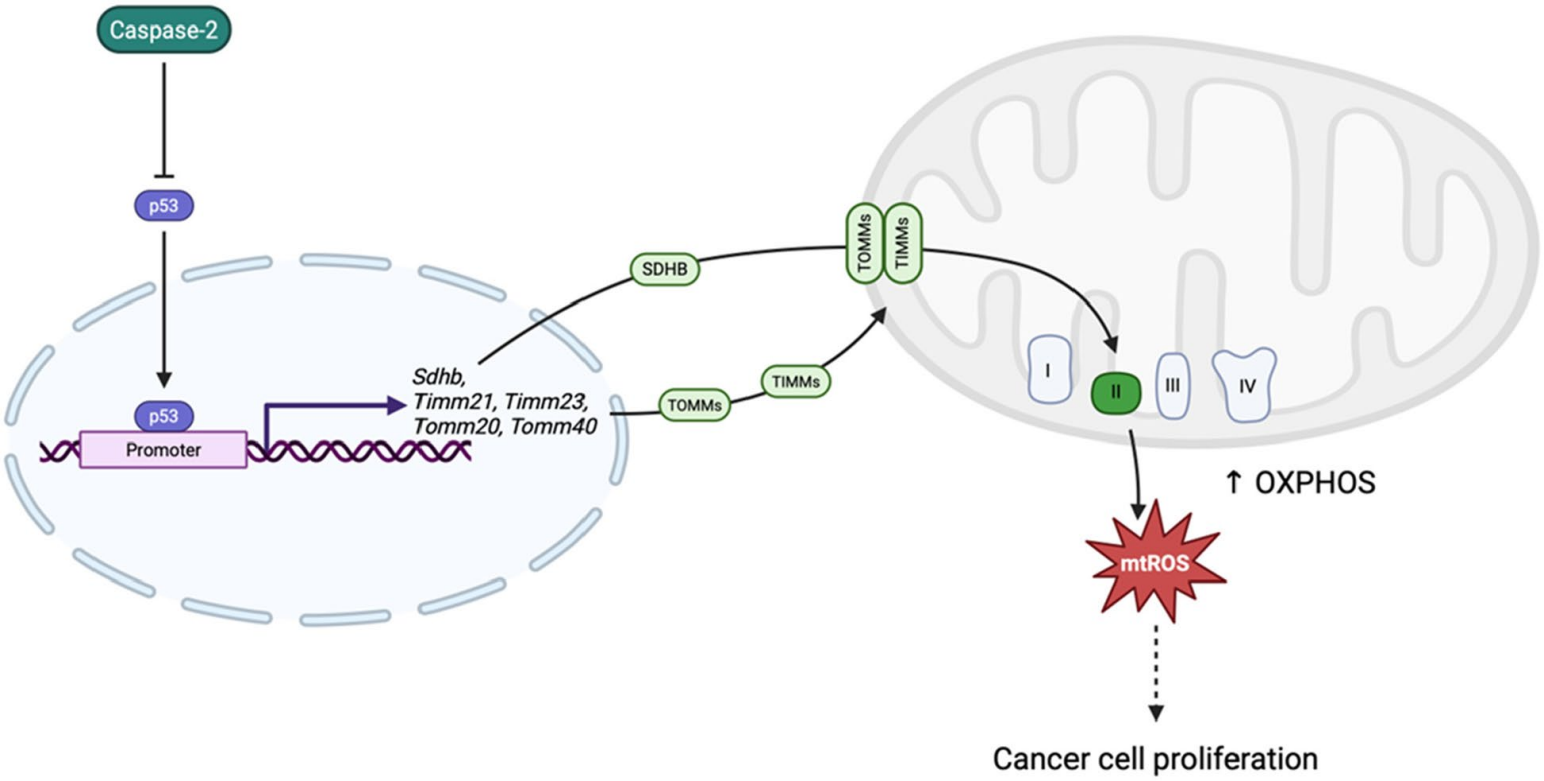
Schematic illustration of the proposed mechanism by which caspase-2 regulates mitochondrial respiratory function

Our study revealed a novel role for caspase-2 in regulating mitochondrial respiratory function in colorectal adenocarcinoma cells. As illustrated in Fig. 7, caspase-2 suppresses p53 expression and downregulates the transcription of mitochondria-related genes, including *Sdhb, Timm21, Timm23, Tomm20*, and *Tomm40*.

This downregulation reduces TIMM–TOMM complex formation, thereby limiting the translocation of SDHB into mitochondria and suppressing mitochondrial complex II activity. However, additional regulatory pathways, such as the JNK/c-Jun signaling axis, may also contribute to the transcriptional control of mitochondrial genes and cannot be excluded. Overall, this study offers new insight into the complex interplay between caspase-2 and mitochondrial function, providing a foundation for future research in this area.

## Supplementary Information

Supplementary Material 1.

## Data Availability

No datasets were generated or analysed during the current study.
