# Supplementary material for: Caspase-2 inhibits mitochondrial respiration in colorectal adenocarcinoma cells
Source: Cell Commun Signal. 2026 Jan 20;24:59. doi: 10.1186/s12964-026-02671-z (PMC12849408; doi:10.1186/s12964-026-02671-z)
Supplement: Supplementary file 1 — Supplementary Material 1. [file 12964_2026_2671_MOESM1_ESM.pdf]

Supplementary data

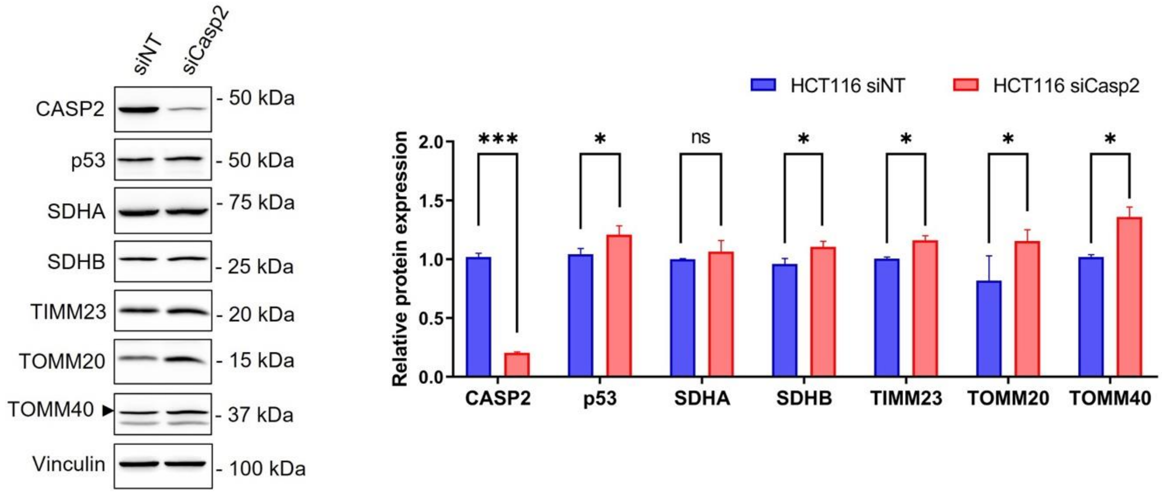

Figure S1. Effects of caspase-2 on mitochondria-related protein content in HCT116 human colon cancer cells. Representative immunoblots and relative protein levels of CASP2, p53, SDHA, SDHB, TIMM23, TOMM20, and TOMM40 in HCT116 cells transfected with siRNA targeting *Casp2* or negative control (NT). Results are shown as the means ± SD of three independent experiments. *$P < 0.05$, **$P < 0.01$, ***$P < 0.001$, ns = not significant.

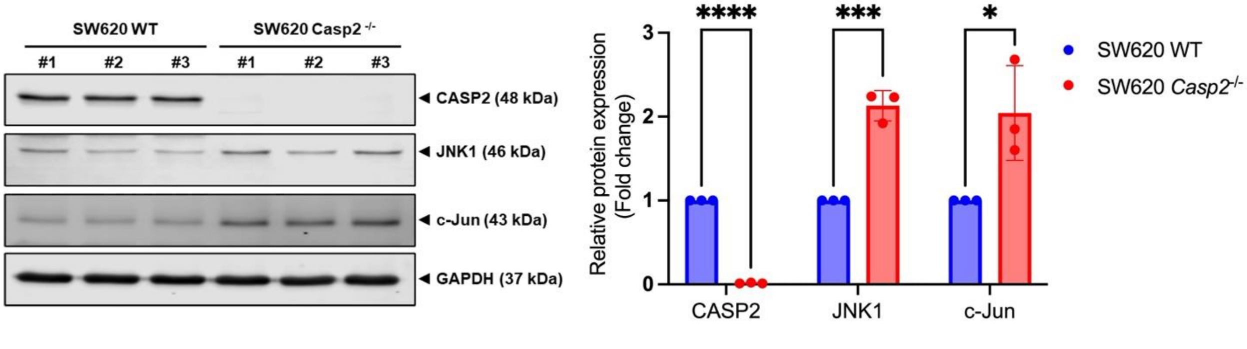

Figure S2. Effects of caspase-2 on JNK1 and c-Jun protein expressions. Representative immunoblots and relative protein levels of CASP2, JNK1, and c-Jun in SW620 WT and SW620 *Casp2*$^{-/-}$ cells. Results are shown as the means ± SD of three independent experiments. *$P < 0.05$, ***$P < 0.001$, ****$P < 0.0001$
